# Alteration of Gut Microbiota by Ketogenic Diet as an Alternative Therapeutic Target for Drug-Resistant Epilepsy

**DOI:** 10.3390/microorganisms13112539

**Published:** 2025-11-06

**Authors:** Mi Tang, Wei Wang, Jiwen Wang, Yingyan Wang

**Affiliations:** 1 Department of Neurology, Shanghai Children’s Medical Center, Shanghai Jiao Tong University School of Medicine, Shanghai 200127, China; gemmattt@sjtu.edu.cn; 2 State Key Laboratory of Genetics and Development of Complex Phenotypes, Department of Microbiology, Fudan Microbiome Center, School of Life Sciences, Fudan University, Shanghai 200438, China

**Keywords:** ketogenic diet, drug-resistant epilepsy, gut microbiota, gut–brain axis, microbiota dysbiosis, neurotransmitters

## Abstract

As one of the most serious and widespread neurological disorders, epilepsy affects nearly 70 million people worldwide. In the development of this disease, significant alterations of gut microbiotas are often observed in the patients. During the treatment of drug-resistant epilepsy, which accounts for ~20–30% of cases, a ketogenic diet (KD), a diet containing high fat, adequate protein, and low carbohydrate, has been widely used and showed promising therapeutic effects. The underlying mechanisms of the neuroprotective effects of a KD have been suggested in recent studies to be connected to the gut microbiota, the composition of which is dramatically influenced by this treatment. In this review, we summarize the recent advances of the relationship between a KD, gut microbiota, and epilepsy, with an emphasis on the gut bacterial changes under KD treatment, hoping to delineate the gut microbiota as a potential therapeutic target in epilepsy.

## 1. Introduction

The human gut microbiota has received immense attention from the fields of microbiology, genetics, and basic and clinical medicine in recent years, and it has been found to play important roles in the immune, endocrine, neurological, and other systems of the human body [1]. The bidirectional signaling network linking the gastrointestinal tract and central nervous system (CNS), known as the gut–brain axis, has been extensively studied for its critical relationship to health and disease [2]. Recently, with the in-depth study of the gut microbiota, the notion has been extended to including the microbiota, termed the “microbiota–gut–brain axis”. The concept associates gut microbiota with various CNS disorders, like epilepsy, Alzheimer’s disease [3], Parkinson’s disease [4], autism spectrum disorder [5], and multiple psychiatric disorders like stress, anxiety, and depression [6]. As the most serious and widespread neurological disorder, epilepsy usually starts in infancy and lasts for an individual’s lifetime. Nearly 70 million people worldwide are affected by it, and approximately~20–30% of these patients are resistant to routine antiepileptic drugs [7]. According to the ad hoc Task Force of the International League Against Epilepsy, those who fail to achieve lasting freedom from seizures after adequate trials with monotherapy or multi-drug combinations of first-line antiepileptic drugs are diagnosed as having drug-resistant epilepsy (DRE) [8]. Interestingly, patients with epilepsy (PWEs) often suffer from gastrointestinal symptoms, and patients with inflammatory bowel disease are more susceptible to epilepsy [9]. A considerable number of studies on gut microbiota and epilepsy have been published, many of which emphasized the significant impacts of dietary intervention. Among these interventions, a ketogenic diet (KD), a high-fat, low-carbohydrate, and adequate-protein diet established early in the 1920s by Wilder [10], has proven efficacy for refractory epilepsy [5,11]. Dramatic compositional changes in gut microbiota in refractory epilepsy patients and the therapeutic effects of KD on epilepsy via regulating gut microecology have been demonstrated in many studies [12,13,14]. However, the gut microbiota’s mechanistic role in the KD’s therapeutic effects to intractable epilepsy has not been fully illustrated. In this review, recent advances regarding the associations of gut microbiota, DRE, and a KD are discussed.

## 2. Gut Microbiota and DRE

### 2.1. Microbiota–Gut–Brain Axis

The human gut microbiota is a complex microbial ecosystem that maintains immune, endocrine, metabolic, and neurological homeostasis [15]. It is estimated that over 100 trillion microorganisms, representing more than 1000 species, inhabit the adult gastrointestinal tract, with Firmicutes and Bacteroidetes constituting more than 90% of the microbial population [16,17,18]. This composition is shaped by numerous internal and external factors, such as diet, medication, age, and lifestyle [19,20]. Among these, diet exerts the most profound and immediate influence, capable of modifying microbial diversity and metabolic functions within days.

The microbiota–gut–brain axis describes the bidirectional communication network connecting the intestinal microbiota and the central nervous system (CNS) [21,22,23]. This interaction occurs through multiple pathways: (1) the neural (vagus nerve) pathway; (2) the neuroendocrine–hypothalamic–pituitary–adrenal (HPA) axis; (3) the immune system; (4) microbially derived neurotransmitters and neuromodulators; and (5) the intestinal mucosal and blood–brain barriers (BBBs) [24,25]. Through these mechanisms, the brain modulates gut motility, secretion, and permeability, while the microbiota regulates neurotransmitter synthesis, neuroinflammation, and neuronal excitability [26,27].

Microbial metabolites such as short-chain fatty acids (SCFAs), bile acids, and tryptophan derivatives play key roles in maintaining BBB integrity, microglial activation, and the balance of excitatory and inhibitory neurotransmission, particularly glutamate and γ-aminobutyric acid (GABA) [27,28]. Disruption of this axis, termed dysbiosis, can result in decreased SCFA production, impaired immune signaling, and altered neurotransmitter metabolism, contributing to the pathogenesis of several neurological disorders, including epilepsy [29,30].

### 2.2. Evidence of Microbiota Dysbiosis in DRE Cases

Vast evidence from animals and human studies has shown that the gut microbiota plays a critical role in brain development and functioning [4,16,29,30]. With the wide use of 16S rRNA sequencing technology, the latest population-based studies revealed not only gut microbiota differences between PWEs and healthy controls (HCs) but also gut bacterial composition variations between individuals with DRE and drug-sensitive epilepsy (DSE, Table 1). These differences between healthy controls and individuals with epilepsy have suggested the possible roles of gut microbes in the pathogenesis of epilepsy. Here, we elaborate on the relationship between the gut microbiota and epilepsy in clinical cases and discuss the modulation of gut microbiota in epilepsy patients.

One of these studies assessed the gut microbe’s diversity in a Turkish cohort with idiopathic focal epilepsy (N = 30) and a HC group (N = 10) by 16s rRNA sequencing in 2018 [31]. Safak et al. [32] analyzed seven main phyla from all samples and unveiled the difference in microbiota at the phylum and species level in the HC group and PWEs. Proteobacteria were found to be higher in PWEs (25.4%) than in HCs (1.5%). Fusobacteria were detected in 10.6% of the PWEs but not in the HCs, while Firmicutes, Bacteroidetes, and Actinobacteria were found to be higher in the HCs than in PWEs. These significant differences in gut microbiota composition suggest that bacterial dysbiosis may play a key role in the etiology of epilepsy. Of note, there was a potentially confounding factor in Safak’s study: the absence of family controls to eliminate the possible effects caused by baseline dietary differences. Another study by Lindefeldt et al. [33] eliminated this confounder by analyzing fecal samples from 12 Swedish children with DRE (aged 2~11 years) and 11 healthy parents, who served as controls, using shotgun metagenomic sequencing. In the patient group, fecal microbiota diversity showed a slight decrease, and the microbiota of children with DRE presented a higher variability. In addition, the relative abundances of Bacteroidetes and Proteobacteria decreased, while both Firmicutes and Actinobacteria increased in children with DRE. The authors also noted that the expression of genes involved in the acetyl-CoA pathway, such as acetyl-CoA acetyltransferase and β-hydroxybutyric-CoA dehydrogenase, were reduced in the patients’ microbiota compared with those in the HCs. Subsequently, following the preliminary analysis of gut microbiota differences between DRE patients and controls, Lindefeldt et al. [33] proceeded to conduct a KD management intervention, which will be discussed in the following section.

Gut microbiota analysis can also be used to determine the difference between drug-sensitive and drug-resistant individuals. The first study of this kind was published in 2018 [34], where Peng and colleagues performed high-throughput 16S rRNA gene sequencing of fecal samples of the participants. After comparing the microbial compositions of DRE patients (N = 42), DSE patients (N = 49), and HCs (N = 65, from the same families as the patients), they found that the DRE group had significantly increased α-diversity compared with the HCs. They also observed that DSE patients and HCs normally had more Bacteroidetes than Firmicutes, while this ratio was reversed in DRE patients. The abundances of specific bacterial genera also increased abnormally [34], including *Clostridium XVIII*, *Akkermansia*, *Atopobium*, *Holdemania*, *Dorea*, *Delftia*, *Coprobacillus*, *Paraprevotella*, and *Fusobacterium.* In addition, the levels of *Lactobacillus* and *Bifidobacteria* were elevated in individuals with fewer seizures (≤4 per year). Meanwhile, the DSE patients displayed comparable community richness and evenness to those of the HCs [34]. These results suggested that dysbiosis might be involved in the pathogenesis of DRE, and the restoration of gut microbiota might be a new method of treatment.

In line with the principle of comparing drug responders and non-responders, Gong and colleagues designed an analysis to display these changes [35]. They performed two independent cross-sectional analyses, including an exploration and a validation cohort, aiming to use the gut microbiota as a biomarker for epilepsy. The exploration cohort contained 55 PWEs and 46 HCs, who were healthy spouses of the patients, and another cohort contained 13 PWEs and 10 HCs. Inclusion and exclusion criteria were similar to the above-mentioned study by Peng et al. [34]. The alpha diversity indexes of the specimens from PWEs were much lower than those from the HCs (*p* < 0.05). Microbiota alterations in PWEs included increases in Actinobacteria and Verrucomicrobia and a decrease in Proteobacteria at the phylum level, and rises in *Prevotella_9*, *Blautia*, *Bifidobacterium*, and others at the genus level [35]. In the subsequent sample analysis, 30 DRE patients were compared to 25 DSE patients in the exploration cohort, and the authors found that DRE patients showed significant enrichment in Actinobacteria, Verrucomicrobia, and Nitrospirae, as well as the genera *Blautia*, *Bifidobacterium*, *Subdoligranulum*, *Dialister*, and *Anaerostipes* [35].

**Table 1:** Representative studies on gut microbiota and epilepsy.

Year	Population (N, Age)	Methodology	Findings	Country	Study
2023	DRE (12, NA) HCs (12, NA)	Fecal samples 16S rRNA sequencing	↑ *Akkermansia muciniphila*, ↑ *Parabacteroides gordonii*	America	Lum et al. [36]
2021	DREPs (20, 41 ± 13.6 years) DSEPs (20, 44 ± 17.2 years)	Fecal samples 16S rRNA sequencing	↔ α-diversity, β-diversity ↑ Firmicutes, *Bifidobacterium*, *Shigella*, *Veillonellales*, *Klebsiella*, *Streptococcus* ↓ Bacteroidetes, *Ruminococcus_g2*, *Bifidobacterium*	Korea	Lee et al. [37]
2020	Exploration cohort: PWEs (55, 15∼50 years), HCs (46, NA)/ DRE (30, NA), DSE (25, NA) Validation cohort: PWEs (13, NA), HCs (10, NA)	Fecal samples 16S rRNA sequencing	↓ α-diversity ↑ Actinobacteria, Verrucomicrobia, *Nitrospirae*, *Blautia*, *Bifidobacterium*, *Subdoligranulum*, *Dialister*, *Anaerostipes* ↓ Bacteroidetes, Proteobacteria	China	Gong et al. [35]
2020	IEPs (8, 1.16–6.92 years), HCs (32, 1.16–6.92 years)	Fecal samples 16S rRNA sequencing	↓ α-diversity ↑ Firmicutes, Actinobacteria, Verrucomicrobia ↓Bacteroidetes, Proteobacteria	Korea	Lee et al. [38]
2020	IEPs (30, 41.3 ± 12.2 years), HCs (10, 31.7 ± 6.8 years)	Fecal samples 16S rRNA sequencing	α-diversity NA ↓ Firmicutes, Bacteroidetes, Actinobacteria, Euryarchaeota ↑ Proteobacteria, Fusobacteria, Spirochaetes	Turkey	Birol Şafak et al. [32]
2019	DREPs (20, 2–17 years), HCs (11, NA)	Fecal samples Metagenomic sequencing	↔ α-diversity ↑ Firmicute, Actinobacteria ↓Bacteroidetes, Proteobacteria	Sweden	Lindefeldt et al. [33]
2018	DREPs (42, 28.4 ± 12.4 years), DSEPs (49, 25.1 ± 14.6 years), HCs (65, 29.4 ± 13.8 years).	Fecal samples 16S rRNA sequencing	↑ α-diversity ↑ Firmicutes, Verrucomicrobiota, *Clostridium XVIII*, *Akkermansia*, *Atopobium*, *Holdemania*, *Dorea*, *Delftia*, *Coprobacillus*, *Paraprevotella*, *Fusobacterium*, etc. ↓ Bacteroidetes	China	Peng et al. [34]

Abbreviations: PWEs, patients with epilepsy; IEPs, idiopathic/intractable epilepsy patients; DREPs, drug-resistant epilepsy patients; DSEPs, drug-sensitive epilepsy patients; HCs, healthy controls; NA, not available. ↑ Increase in abundance. ↓ Decrease in abundance. ↔ No significant changes in abundances.

Considering the inconsistent results of the published studies regarding the gut microbiota as a diagnostic biomarker of DRE that had been published at that time, Lee et al. [38] analyzed fecal samples from a population of 8 Korean children aged 1~7 years old with intractable epilepsy and 32 age-matched HCs. Using the 16S rRNA gene sequencing approach, they found that α-diversity was higher in epilepsy patients, and β-diversity showed a clear difference in bacterial composition between the two groups. In the epilepsy group, the amount of Bacteroidetes was lower and the amount of Actinobacteria was higher than in the healthy group. Species biomarkers for intractable epilepsy included the *Enterococcus faecium* group, *Bifidobacterium longum* group, and *Eggerthella lenta*. By analyzing those data, the authors confirmed that patients with intractable epilepsy did, indeed, have gut bacterial dysbiosis. The following year, Lee and colleagues [37] conducted another exploratory trial based on adult patients at their clinic. They prospectively included 44 adult epilepsy patients and classified them into drug-responsive and drug-resistant groups but found no differences in α or β diversity between the two groups. While the abundances of Firmicutes, *Bifidobacterium*, *Shigella*, *Veillonellales*, *Klebsiella*, and *Streptococcus* increased in DRE patients, the relative abundances of *Bacteroides*, *Ruminococcus_g2*, and *Bifidobacterium* were augmented in DSE patients. The significant difference in the composition of gut microbiota among patients with DRE and DSE supported the conclusions in Peng and Gong’s research.

Most recently, Lum et al. [36] conducted a dual human–mouse study in a U.S. pediatric cohort to investigate microbial dysbiosis in epilepsy. The authors observed that children with DRE exhibited lower microbial diversity but enrichment of *Akkermansia muciniphila* and *Parabacteroides gordonii*. Importantly, fecal microbiota transplantation from DRE patients into germ-free mice significantly increased seizure thresholds and reduced seizure frequency, confirming that the gut microbiota directly modulates seizure susceptibility. These findings provided the first causal evidence in humans and mice linking microbiota alterations to seizure control.

Taken together, these six clinical studies assessed the diversity and composition of gut microbiota in PWEs and suggested the existence of microbial dysbiosis in DRE patients, indicating the potential value of using the gut microbiota as a sensitive biomarker for diagnosis and a treatment target to enhance control of seizures. Notably, most studies showed that α-diversity in the HC group was higher than that in the DRE group [32,33,35,37,38]. However, Peng’s study demonstrated an opposite trend, with increased α-diversity in the DRE group [34], which indicated that the alteration of microbiota in epilepsy patients might not always be consistent. Considering the influence of study design, age, diet, living environment, and other impacts on gut microbes, a larger-sample analysis based on reasonable control variables is still needed.

## 3. Links Between KD and Gut Microbiota in DRE

### 3.1. Gut Microbiota and KD

Unlike the host genome, the gut microbiota exhibits a considerable plasticity and can be readily influenced by a variety of stimuli, including diet [31], medication [39], stress [40], infection [41], and lifestyle factors [42]. Among these, diet represents the dominant determinant shaping the gut microbiota’s composition and metabolic activity. Numerous studies have shown that daily dietary habits are pivotal in defining microbial diversity across different health states and life stages [31]. In line with Hippocrates’ ancient notion that “food is medicine,” modern evidence confirms that diet-induced changes in microbial ecology can profoundly affect disease susceptibility, either by triggering dysbiosis or restoring microbial balance [43].

Dietary composition primarily consists of carbohydrates, fiber, proteins, and fats, each exerting distinct influences on the gut microbial community [43]. The impact of different types of diet on gut microbiota profiles and the pathogenesis of illness related to this have been studied extensively [44]. For instance, the Western diet, providing 40–55% of calories from fats [45], is typically high in saturated fats and refined sugars. Such diets have been consistently associated with inflammation, impaired intestinal barrier function, and reduced production of SCFAs [46,47]. On the other hand, microbiota-accessible carbohydrates (MACs)—abundant in dietary fiber—play essential roles in maintaining microbial diversity, supporting SCFA production, and promoting gut–brain axis homeostasis [48,49,50]. MAC-deficient diets, however, sometimes exacerbate inflammatory diseases such as allergies, infections, and autoimmunity [46,51]. Another example is the Mediterranean diet, which is rich in omega-3 fatty acids and polyphenols, increases anti-inflammatory bacteria, and is linked to reduced obesity, cancer, and chronic inflammation [52,53]. Dietary protein also critically influences microbial metabolism, as it provides nitrogen, which is essential for bacterial growth and the synthesis of SCFAs [44,54]. However, excessive protein intake may increase harmful fecal metabolites such as hydrogen sulfide, trimethylamine, and phenols, contributing to inflammatory bowel disease and cancer [55].

Building on this evidence, the KD—a high-fat, low-carbohydrate regimen—represents a well-defined nutritional model that profoundly alters the gut microbiota’s structure and function. A classic KD typically follows a 3:1 or 4:1 ratio, meaning that 80–90% of total energy is derived from fats, while only 10–20% comes from carbohydrates and proteins [56]. Although excessive fat intake has been known to have adverse effects [45,47], modern KD protocols have been refined for improved safety and compliance. Four primary KD variants are commonly employed in epilepsy therapy, as summarized in Table 2: the classic KD, medium-chain triglyceride (MCT) diet, modified Atkins diet, and low-glycemic-index therapy [57].

Originally introduced in the 1920s for treating refractory epilepsy [10], the KD has since been extended to other neurological and metabolic disorders, including migraine [58], glaucoma [56], Alzheimer’s disease [59], obesity [60], cancer [44], and respiratory disorders [61]. Although its clinical efficacy in reducing seizures is well established, the precise mechanisms underlying KD’s antiepileptic effects remain incompletely understood. Current evidence suggests that increased ketone body utilization and polyunsaturated fatty acid metabolism play crucial roles [57,62], potentially involving regulation of brain energy metabolism [63], ion-channel activity [64], neurotransmitter synthesis [65], epigenetic modification [66], and gut microbiota modulation [67].

A KD markedly decreases microbial diversity due to its extremely low carbohydrate content, resulting in reduced fiber-degrading bacteria [68]. The microbial community typically shifts toward increased *Bacteroides* and *Prevotella* populations, with an approximately 50% reduction in *Escherichia coli* levels [14]. In a mouse model of refractory epilepsy induced by 6 Hz stimulation, both germ-free and antibiotic-treated mice failed to achieve seizure protection under a KD, whereas fecal microbiota transplantation from KD-treated donors restored the antiseizure effect [67,69]. These studies provide compelling evidence that gut microbiota are essential mediators of KD efficacy.

Further human KD intervention studies have identified responder- and non-responder-specific microbial signatures [70]. Differences were observed at the phylum, family, and genus levels, as well as in the production of microbial metabolites. Such findings suggest the presence of ketone-sensitive microbiota within the gastrointestinal tract that are critical for seizure control [69,71]. These “keto microbiota,” along with their metabolites, can synthesize inhibitory neurotransmitters, such as GABA, by regulating metabolic precursors [72]. Together, these observations indicate that KD exerts its antiepileptic effects through a microbiota–metabolite–neurotransmitter regulatory pathway, modulating neuronal excitability and seizure susceptibility [69].

### 3.2. KD Intervention Studies and Microbiota-Mediated Mechanisms

As discussed above, clinical analyses have demonstrated distinct alterations in the gut microbiota of patients with DRE. With growing interest in the microbiota–gut–brain axis, multiple studies have investigated how a KD reshapes gut microbial composition and how these changes may contribute to seizure control.

A landmark study by Olson et al. [67] first established the causal link between the KD and seizure protection mediated by gut microbiota. Using two mouse models of 6 Hz induced refractory epilepsy, they found that antibiotic-treated KD-fed mice exhibited increased seizure susceptibility due to microbiota depletion. Remarkably, recolonization with normal gut microbiota restored seizure protection. Feding with a KD reduced microbial diversity but markedly increased the relative abundance of *A. muciniphila*. Co-colonization of *A. muciniphila* and Parabacteroides prevented seizures in germ-free mice, whereas single-species colonization failed to do so. Subsequent analyses revealed an elevated hippocampal GABA/glutamate ratio in KD-fed mice, suggesting a mechanistic link between microbial metabolism and neurotransmitter modulation. This pivotal study provided the first direct evidence that gut microbiota are required for the antiseizure effects of KD and that specific bacterial interactions regulate peripheral metabolites influencing hippocampal neurotransmission [69].

Subsequent human studies corroborated these findings. Xie et al. [14] analyzed fecal microbiota from 14 children with refractory epilepsy and 30 healthy controls using 16S rRNA sequencing. After one week of KD treatment, Bacteroidetes increased, whereas overall richness and Proteobacteria declined. At the genus level, Cronobacter decreased, while Bacteroides, Bifidobacterium, and Prevotella increased. Since Bacteroides are involved in lipid metabolism and cytokine regulation (IL-6, IL-17) [73], their increased abundance may reflect enhanced anti-inflammatory potential associated with seizure reduction [74]. These results indicated that a KD could alleviate seizure symptoms by reshaping gut microbiota composition in pediatric patients [14].

In a prospective trial of 20 children with intractable epilepsy, Zhang et al. [70] applied a 4:1 KD for six months. Half of the participants achieved ≥50% seizure reduction. Responders displayed lower alpha diversity and a significant shift from Firmicutes toward Bacteroidetes, along with enrichment of *Alistipes*, *Lachnospiraceae*, *Ruminococcaceae*, and *Rikenellaceae*. These taxa differences correlated with treatment efficacy, suggesting that specific bacterial signatures could serve as biomarkers and therapeutic targets for DRE.

Similarly, Lindefeldt et al. [33] performed shotgun metagenomic sequencing on fecal samples from 12 children with DRE before and three months after KD treatment. A KD did not change alpha diversity but significantly altered microbial species and metabolic pathways. *Bifidobacterium*, *Eubacterium rectale*, and *Dialister* decreased, while Escherichia coli increased markedly. Functional profiling revealed 29 altered SEED subsystems, including decreases in 7 carbohydrate metabolism pathways, indicating a major metabolic reprogramming of gut microbiota. These findings highlight Bifidobacteria and E. coli as key contributors to KD-induced metabolic remodeling.

Further insights were provided by Gong et al. [75], who conducted a cross-sectional study involving 12 children with DRE and 12 healthy controls. After six months of KD therapy, fecal SCFA concentrations increased and correlated positively with beneficial microbial taxa. Specifically, *Actinobacteria*, *Bacteroides*, *Blautia*, *Anaerostipes*, and *Subdoligranulum* were enriched, whereas potentially pro-convulsive genera declined. These results demonstrate that KD can modulate both microbial composition and metabolite profiles, supporting its capacity to restore gut–brain axis homeostasis in epilepsy.

In addition to these earlier studies, Li et al. [76] provided further mechanistic insights using a rat model of epilepsy treated with a 3:1 ratio KD. Combining 16S rRNA sequencing with metabolomic profiling, the authors revealed increased abundance of *Lactobacillus* and *Akkermansia* and decreased levels of *Bacteroides* following KD administration. These microbial shifts were accompanied by elevated concentrations of SCFAs, including butyrate and propionate, and improved mitochondrial function and oxidative stress parameters. This study highlighted the potential role of SCFA-producing bacteria and improved redox homeostasis as key mediators of the KD’s antiseizure effects.

Building upon these findings, Özcan et al. [77] investigated how dietary fiber content modulates the efficacy of KD in both clinical and experimental models. They compared standard and high-fiber KD formulations and found that the inclusion of microbiota-accessible carbohydrates enriched *Akkermansia*, *Roseburia*, and *Bacteroides*, taxa that are known for SCFA and anti-inflammatory metabolite production. Mice and human subjects receiving a high-fiber KD exhibited greater seizure protection, reduced neuroinflammatory markers, and enhanced SCFA concentrations. These results demonstrated that gut microbiota-derived metabolites, particularly SCFAs, contribute to the neuroprotective effects of KD and that dietary fiber acts as a crucial modulator of KD efficacy.

Collectively, these studies provide convergent evidence that the ketogenic diet (KD) reshapes gut microbiota diversity and functionality, thereby contributing to seizure control. The key methodologies, inferences, and major findings are summarized in Table 3. Despite substantial progress, the precise mechanisms linking microbial alterations to seizure modulation remain incompletely understood. Proposed mechanisms include regulation of cerebral energy metabolism; modulation of neurotransmitters such as GABA, glutamate, and aspartate; and effects on neuronal excitability and synaptic transmission [57].

The integrated mechanistic relationships between specific gut microbes, their metabolites, and seizure modulation under a KD are summarized in Table 4. Taken together, these findings indicate that a KD promotes the enrichment of short-chain fatty acid (SCFA)- and bile acid-producing taxa (*Akkermansia*, *Parabacteroides*, *Eubacterium*), while reducing saccharolytic bacteria such as *Bifidobacterium* and *Lactobacillus*. These microbial and metabolic shifts contribute to strengthened blood–brain barrier (BBB) integrity, reduced neuroinflammation, and elevated seizure thresholds [36,67,76,77,78,79,80].

Future research should aim to elucidate how specific bacterial taxa and their metabolites influence neuronal membrane potential and neurotransmitter balance. A better understanding of these microbiota–metabolite–neurotransmitter pathways will refine mechanistic models of the KD’s efficacy and facilitate the development of microbiota-targeted interventions for refractory epilepsy.

## 4. Conclusions

**Figure 1:**
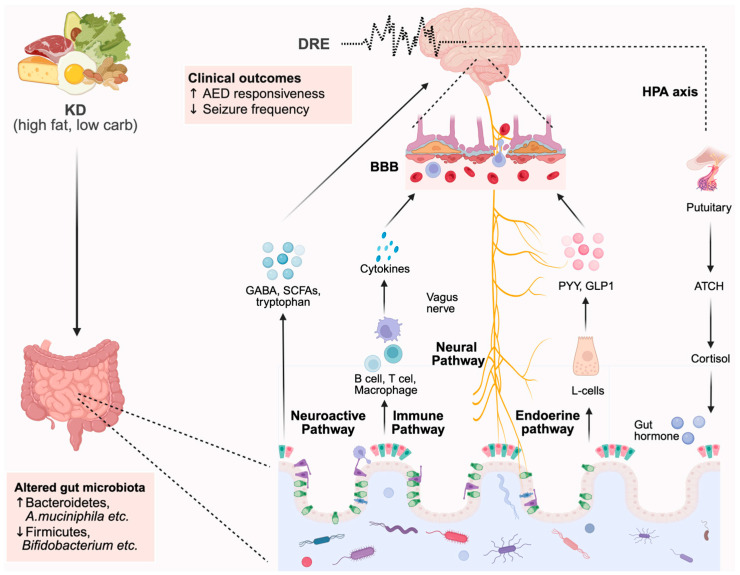
Integrated model illustrating microbiota–metabolite–neural pathways linking ketogenic diet to seizure modulation.

Over the past decade, gradually emerging evidence has supported a crucial connection between the microbiota–gut–brain axis and epilepsy. Gut microbes appear to be key modulators of central nervous system signaling, particularly in DRE treated with the KD. Both animal and human studies suggest that the KD’s antiepileptic effects may depend, at least in part, on microbiota-mediated mechanisms. In this review, we summarize recent murine experimental data and clinical evidence to explore the association between gut microbiota, the KD, and DRE (as schematically summarized in Figure 1). Based on these findings, it is reasonable to speculate that gut microbiota dysbiosis may be a strong factor in both the development and severity of epilepsy. Notably, our recent metagenomic and bioinformatic analyses revealed that the abundance of certain bacteria, such as *A. muciniphila* and *Parabacteroides gordonii*—previously identified as antiepileptic species by Olson et al. [67]—increased dramatically following KD treatment, consistent with their demonstrated capacity for lipid utilization in vitro [81]. However, for other bacteria, abundance and growth rates were not correlated, suggesting that KD-induced changes in microbial composition may also be driven by non-nutritional factors.

These findings deepen our understanding of how the KD reshapes the gut ecosystem to exert its therapeutic effects. Nonetheless, several critical questions remain unresolved, including the multifaceted mechanisms by which microbial metabolites influence seizure susceptibility and how microbiota can be leveraged as therapeutic targets. To address these gaps, future studies should adopt multi-omics strategies, integrating metagenomics, metabolomics, and transcriptomics, to more precisely identify key microbial taxa, metabolic pathways, and bioactive metabolites associated with seizure control.

Importantly, translating these findings into clinical practice will require exploration of microbiota-oriented interventions, such as probiotics, prebiotics, and personalized dietary approaches, as adjunctive strategies to enhance the efficacy of the KD in DRE management. A systems-level understanding of host–microbe interactions will provide new insights into the pathogenesis of epilepsy and the development of microbiome-based precision therapies.

## Figures and Tables

**Table 2:** Compositions of the four types of KD.

KD Type	Fat (g)	Protein (g)	Carbohydrate (g)	Fat Calories (% of Total)
Classic ketogenic diet	100	17	8	90
Medium-chain triglyceride diet	78	25	50	70
Modified Atkins diet	70	60	10	70
Low-glycemic-index therapy	70	40	40	45

**Table 3:** Gut microbiota alterations after KD in DRE.

Subjects	Subject Characteristics	Duration	Type of KD	Methodology	Gut Microbiota Alterations	Study
Mice	6 HZ induced seizure model of RE	2 weeks	6:1 ratio KD	16S rRNA gene amplicon sequencing	↓ α-diversity; ↑ *A.muciniphila*, *Parabacteroides*, *Hippocampal* ↓ *Allobaculum*, *Bifidobacterium*, *Desulfovibrio*	2018 Olson et al. [67]
Humans	14 DREPs vs. 30 HCs	1 week	2:1 ratio KD	16S rRNA gene amplicon sequencing	↑ Bacteroidetes ↓ Proteobacteria, *Cronobacter*	2017 Xie et al. [14]
Humans	20 DREPs	6 months	4:1 ratio KD	16S rRNA gene amplicon sequencing	↓ α-diversity ↑ Bacteroidetes ↓ Firmicutes In the non-responsive group: ↑ Clostridiales, Rikenellaceae, Ruminococcaceae, Alistipes, Lachnospiraceae	2018 Zhang et al. [70]
Humans	12 DREPs vs. 11 HCs	6 months	4:1 in 7; 3.5:1 in 2; 3:1 in 3 patients	Shotgun metagenomic DNA sequencing	↔ α-diversity ↑ Proteobacteria (*E. coli*) ↓ Actinobacteria, *Dialister*, *Bifidobacteria*, and *E. rectale*	2019 Lindefeldt et al. [33]
Humans	12 DREPs vs. 12 HCs	6 months	NA	16S rRNA gene amplicon sequencing	↑ *Subdoligranulum*, *Dialister*, *Alloprevotella* ↓ *Bifidobacterium*, *Akkermansia*, *Enterococcaceae*, *Actinomyces*	2021 Gong et al. [75]
Rats	PTZ-induced epilepsy model under KD feeding	4 weeks	3:1 ratio KD	16S rRNA gene amplicon sequencing Shotgun metagenomic DNA sequencing	↑ *Lactobacillus*, *Akkermansia* ↓ *Bacteroides*	2024 Li et al. [76]
Mice	Mice (n = 48, 12 per group) fed KDs with different fiber contents	8 weeks	Modified KD	16S rRNA gene amplicon sequencing Shotgun metagenomic DNA sequencing	↑ *Akkermansia*, *Roseburia*, *Bacteroides*	2025 Özcan et al. [77]

Abbreviations: RE, refractory epilepsy; DREPs, drug-resistant epilepsy patients; HCs, healthy controls; NA, not available. ↑ Increase in abundance. ↓ Decrease in abundance. ↔ No significant changes in abundance.

**Table 4:** Mechanistic links between specific gut microbes or metabolites and seizure modulation in ketogenic diet.

Microbial Taxa Change Under KD	Key Metabolites or Pathways	Host Targets and Mechanisms	Physiological/Neurological Effects	Evidence Type	Key Findings/Reference
↑ *A. muciniphila*	SCFAs (acetate, butyrate), mucin degradation	↑ Tight junction proteins ↑ BBB integrity ↓ Pro-inflammatory cytokines	↓ Neuroinflammation ↑ Seizure threshold	Mouse KD model [67]	Restoration of BBB and protection against seizures via SCFA-dependent signaling
↑ *Parabacteroides* spp.	GABA, bile acid metabolism	↑ GABA ↓ Glutamate	↓ Neuronal excitability Improved seizure control	Mouse and human samples [36]	KD responders showed enrichment of Parabacteroides correlating with seizure reduction
↓ *Bifidobacterium*, *Lactobacillus*	Carbohydrate fermentation, lactate	Reflects carbohydrate restriction; altered energy substrate utilization	Facilitates ketosis and sustained β-hydroxybutyrate production	Clinical KD cohort [76]	KD-induced reduction in saccharolytic bacteria supports stable ketone production
↑ *Clostridium* spp.	Deoxycholic acid, lithocholic acid	Activates FXR/TGR5 receptors	↓ Microglial activation Anticonvulsant effect	Preclinical studies [79]	Secondary bile acids mediate anti-inflammatory neuroprotection
↑ *Desulfovibrio* spp.	Hydrogen sulfide	Modulates oxidative stress and mitochondrial function	↓ ROS accumulation neuroprotection	Mouse KD model [80]	KD enriches Desulfovibrio species that regulate redox homeostasis
↑ *Eubacterium and Blautia* spp.	Butyrate, acetate	Enhances GABAergic signaling; supports gut–brain axis	↑ GABAergic tone ↓ Hyperexcitability	Human pilot KD study [77]	Butyrate-producing taxa correlate with improved seizure control
Overall KD-induced microbial pattern	↑ SCFAs ↑ Bile acids ↓ Inflammatory cytokines	BBB integrity, immune modulation, neurotransmitter synthesis	↑ Seizure threshold ↓neuroinflammation ↑ AED responsiveness	Integrated multi-omics evidence [78]	KD–microbiome interaction mediates metabolic and neuronal stabilization

Abbreviations: KD, ketogenic diet; SCFA, short-chain fatty acid; BBB, blood–brain barrier; AED, antiepileptic drug; FXR, farnesoid X receptor; TGR5, Takeda G protein-coupled receptor 5; ROS, reactive oxygen specie.↑ Increase in abundance. ↓ Decrease in abundance.

## Data Availability

No new data were created or analyzed in this study. Data sharing is not applicable to this article.

## Abbreviations

The following abbreviations are used in this manuscript:

KD	ketogenic diet
CNS	central nervous system
DRE	drug-resistant epilepsy
PWEs	patients with epilepsy
IEPs	idiopathic/intractable epilepsy patients
DREPs	drug-resistant epilepsy patients
HCs	healthy controls
RE	refractory epilepsy
